# Characteristics and possible mechanisms of metabolic disorder in overweight women with polycystic ovary syndrome

**DOI:** 10.3389/fendo.2022.970733

**Published:** 2023-01-12

**Authors:** Jin Yu, Yulai Zhou, Jie Ding, Danying Zhang, Chaoqin Yu, Hefeng Huang

**Affiliations:** ^1^ International Peace Maternity and Child Health Hospital, School of Medicine, Shanghai Jiao Tong University, Shanghai, China; ^2^ Department of Gynecology of Traditional Chinese Medicine, Changhai Hospital, Naval Medical University, Shanghai, China; ^3^ Hospital of Obstetrics and Gynecology, Fudan University, Shanghai, China

**Keywords:** polycystic ovary syndrome, overweight, metabolism, inflammation, insulin resistance

## Abstract

**Background:**

Polycystic ovary syndrome (PCOS) is a kind of endocrine and metabolic disorder, disturbing the females of reproductive age. Here, we aimed to investigate the metabolic characteristics of overweight women with PCOS and analyze the possible mechanisms.

**Methods:**

We conducted a cross-sectional study on 947 patients with PCOS, who were classified according to body mass index (BMI) as overweight (BMI ≥ 24 kg/m^2^) or non-overweight (BMI ≤ 23.9 kg/m^2^). The clinical symptoms, endocrine features, metabolic status, and inflammatory levels of the patients were comprehensively assessed and compared between the patients of the two groups. Additionally, a predictive study on the correlation between inflammation and metabolism was performed using STRING and Cytoscape software, and the possible mechanisms of metabolic disorders involved in the overweight PCOS were preliminarily explored.

**Results:**

Overweight PCOS was associated with increased average age, waist-to-hip ratio, and the incidence of acanthosis nigricans. These patients were susceptible to familial hypertension and diabetes, and exhibited evident characteristics of low levels of luteinizing hormone (LH) and the ratio of LH to follicle-stimulating hormone, and were more inclined to insulin resistance (IR). Furthermore, overweight PCOS presented with a chronic low-grade inflammation state with increased levels of inflammatory cytokines complement components C5/C5α, CXCL12/SDF-1, MIF, and Serpin E1/PAI-1 evidently compared with those in non-overweight PCOS. Pearson analysis showed that these inflammatory cytokines were directly or indirectly correlated with IR. The STRING and Cytoscape network analysis predicted that inflammatory cytokines CXCL12/SDF-1, Serpin E1/PAI-1 and MIF might be crucial for inducing IR in overweight PCOS women through various biological functions and signal transductions including the JAK-STAT cascade, ATP biosynthesis, and HIF-1 signaling.

**Conclusions:**

Overweight patients with PCOS are prone to low gonadal levels, IR, and chronic low-grade inflammation. Inflammatory cytokines CXCL12/SDF-1, Serpin E1/PAI-1and MIF might lead to IR through multiple biological functions and signal transductions in overweight PCOS.

## 1 Introduction

Polycystic ovarian syndrome (PCOS), one of the most prevalent and complicated endocrine disorders, affects up to 1 in every 5-6 reproductive-aged women (1, 2). It increases a wide range of risks including infertility, angiocardiopathy, metabolic syndrome (e.g., dyslipidemia and type 2 diabetes), and psychological features (e.g., depression, anxiety, and low quality of life). PCOS is highly heterogeneously characterized by hyperandrogenism and evidence of ovarian dysfunction, such as chronic oligoanovulation or micropolycystic morphology of the ovary, in accordance with the Rotterdam consensus criteria; however, up to 75% of PCOS women remain reportedly undiagnosed (3).

The pathogenesis of PCOS is heterogeneous and has not yet been fully understood, despite numerous studies being conducted. Notably, androgen excess, insulin resistance (IR), and chronic low-grade inflammation are the most critical risk factors; they may interact in a vicious cycle in the physiological pathogenesis of PCOS (4). Rocha et al. reported that inflammatory and metabolic derangements coexisted in PCOS, which are further fueled by androgen excess (5). Shorakae et al. believed that chronic low-grade inflammation might play a mediating role in the relevance of IR and hyperandrogenism potentially (6). However, the exact pathogenesis of PCOS and the inter-associations of these components remain enigmatic.

IR and the associated hyperinsulinemia, usually clinically manifested as overweight or obesity, have been shown to induce both the endocrine and reproductive traits of PCOS. Although it is not part of the diagnostic criteria, the current guidelines gradually emphasize the pivotal role of IR in PCOS. Recently, growing evidence has indicated a high prevalence of PCOS in women with IR (7), while research revealed that approximately three-quarters of women with PCOS have impaired insulin sensitivity and about one-third of these women have metabolic syndrome (8). The relationship between PCOS and IR has been consistently studied using the glucose clamp methodology (9). However, the causes and effects of this dangerous association and the underlying mechanisms remain unelucidated, and these research topics have attracted the attention of scholars. Thus, a cross-sectional study and a network data predictive analysis research were conducted in this study, aiming to provide guidance for the clinical diagnosis and treatment of PCOS by identifying the relationship between metabolic disorders and inflammatory status in overweight women with PCOS and exploring the possible mechanisms between inflammatory factors and IR.

## 2 Methods and materials

### 2.1 Clinical trial design

We reviewed the medical records of all women who visited the outpatient department of the First Affiliated Hospital of Naval Military Medical University from 2016 to 2020 in Shanghai, China. The study protocol was appraised by the Chinese Ethics Committee of Registering Clinical Trials (Ethics No. ChiECRCT-20160050).

#### 2.1.1 Inclusion and exclusion of PCOS cases

The diagnosis of each woman with PCOS was conducted based on the guidelines of the Rotterdam ESHRE/ASRM Consensus: rare ovulation or anovulation (defined as menstrual cycles > 35 days or < 21 days); clinical manifestations of hyperandrogenism or biochemical hyperandrogenism; and polycystic ovary morphology (defined as 12 or more (2-9 mm) follicles in each ovary identified *via* transvaginal ultrasonography). We included cases of patients who complied with at least two of the aforementioned diagnostic criteria, excluding cases of any major systemic illness, congenital adrenal hyperplasia, diabetes, prolactinemia, acromegaly, Cushing’s syndrome, androgen-secreting neoplasm, and clinical and laboratory features indicating other pituitary or adrenal disorders.

#### 2.1.2 Data collection of baseline information and anthropometric measurements

Baseline information such as age, disease course, and family histories (primarily covering grandparents, maternal grandparents, parents, and siblings) including high blood pressure, diabetes, menstrual irregularity, and infertility, of the patients with PCOS were collected by the nurses, residents, and clinicians during routine hospital visits. Additionally, anthropometric indicators, including body mass index (BMI) and waist-to-hip ratio (WHR), were measured and calculated according to the tenet of our study. BMI was determined by dividing weight (kg) by height squared (m^2^). As per the Chinese Standard Consensus Statement of BMI (10), patients were categorized as overweight at a BMI ≥ 24 kg/m^2^ and non-overweight at a BMI ≤23.9 kg/m^2^. The WHR was calculated by dividing waist circumference (cm), by central obesity was defined at WHR>0.8 (11).

#### 2.1.3 Assessment of clinical symptoms

The following clinical symptoms of all enrolled patients with PCOS were observed: menstruation (the menstrual cycle and length of menstruation), acne (based on the Rosenfield scoring system), hirsutism (based on the Ferriman-Gallwey scoring system), and acanthosis nigricans (refers to the darkening, thickening, and roughness of the skin in the neck, armpits, groin, and other skin folds). These symptoms were comprehensively evaluated by two or more professional clinicians.

#### 2.1.4 Detection of endocrine and metabolic parameters

Fasting blood samples were obtained on 2-5 days of the menstrual cycle (or progesterone withdrawal bleeding) in the mornings; the serum samples were collected in 10 mL vacutainer tubes, centrifuged, and stored at -80°C prior to analysis. The serum levels of luteinizing hormone (LH), follicle-stimulating hormone (FSH), estrogen (E2), testosterone (T), prolactin (PRL), and dehydroepiandrosterone sulfate (DHEAS) were determined using chemiluminescence immunoassay, while that of fasting blood glucose (FBG) was quantified using the hexokinase method. The oral glucose insulin-releasing test (OGIRT) was conducted with 75 g of glucose. Blood samples were obtained, and the serum insulin levels at 30, 60, 90, 120, and 180 min during the OGIRT were measured using electrochemiluminescence. These measurements were performed in the Clinical Laboratory Department of the First Hospital Affiliated to Naval Medical University (Reagent kits used were provided in **Supplementary Table 1**). Inter- and intra-assay coefficients of variation were <5%. Additionally, the IR of patients was assessed using the following three indicators: The area under the insulin curve (IAUC) was calculated according to the formula IAUC = 0.5 × (INS_0min + INS_180min) + 0.75 × INS_60min + INS_30min + INS_120min; INS resistance index (HOMA-IR) was calculated based on the formula HOMA-IR = fasting insulin (FINS, INS_0min, µU/L) × FBG (mmol/L)/22.5; and INS sensitivity index (ISI) was calculated according to the formula ISI = 1/(FBG × INS_0min).

#### 2.1.5 Verification of serum inflammatory cytokine protein microarray detection and ELISA verification

Eight adult patients with PCOS were randomly selected from overweight and non-overweight groups, and the fasting venous blood serum of these women was obtained. Cytokine arrays were used to determine the relative levels of different cytokines and chemokines. As previously described (12–14), the serum inflammatory array in our research was performed using the protocol provided by the manufacturer of the Proteome Profiler Human Cytokine Array Kit (R&D Systems). Briefly, the nitrocellulose membranes containing captured antibodies against 36 different human cytokines were incubated with the serum samples (200 µL of each) overnight at 4°C. The membranes were washed before the addition of detection antibody for 1 h and streptavidin-HRP for 30 min. After development, the films were scanned, and the images were quantified by using Image J (National Institutes of Health, USA). The data analysis was performed by the Shanghai Uninvest Biotechnology Company, and the special inflammatory cytokines (having significant differences between the two groups) were screened. Furthermore, another 60 patients with PCOS (30 in each group) were randomly selected, and the significantly different inflammatory cytokines between the overweight and non-overweight PCOS groups were verified using ELISA (Beyotime systems). Details of the reagent kits used were provided in **Supplementary Table 1**.

### 2.2 STRING and Cytoscape network analysis

STRING is a database for predicting protein-protein interaction (PPI) with confidence score ranges (minimum required interaction score: medium 0.4; max number of interactors to show: 1^st^ shell no more than 10 interactors) (15). The target proteins were selected with species limited to “Homo sapiens” and a confidence score > 0.4 (16). The direct or indirect interactions with specific inflammatory cytokines detected using protein microarray and the IR-related functional proteins acquired from the KEGG database (https://www.kegg.jp/) were obtained through STRING (https://cn.string-db.org/). Subsequently, the results of the PPI network analysis were imported into Cytoscape 3.8.0, and the degree value of the specific inflammatory cytokines was calculated using the analysis plug-in of the software (the higher the Degree Value, the greater the correlation). Lastly, a direct interaction network between the specific inflammatory cytokines and key IR-related functional proteins was reconstructed by Cytoscape. Moreover, the bioinformatic analysis between these crucial proteins was performed using the OECloud tools at https://cloud.oebiotech.cn.

### 2.3 Statistical analyses

The SAS 9.4 statistical software was used for data analyses. Continuous variables are presented as mean ± standard deviation, and categorical variables are described as numbers (percentages). The Student’s t-test was applied for comparisons of the mean between the two groups when the variables were normally distributed, and the Pearson correlation was used for the correlation analyses. The F-test was utilized for the equality of variance test. Fisher’s exact test was applied when the number of categorical variables was less than 5 in each group and Satterthwaite was applied when the continuous variables were skewed. Statistical significance was set at P< 0.05, and all tests were two-tailed.

## 3 Results

Medical records of 947 PCOS women were reviewed and included in this study. The included patients with PCOS were aged 13-42 years and had a disease course of 0.25-21 years. Based on the BMI of patients, 246 and 701 women with PCOS were enrolled in the overweight and non-overweight groups, respectively. The prevalence of overweight, central obesity (WHR > 0.8), paramenia, acne (Rosenfield score > 0), hirsutism (Ferriman-Gallwey score > 9), and acanthosis nigricans (positive) was found to be 25.98%, 59.13%, 98.42%, 74.06%, 51.12%, and 40.00%, respectively. Furthermore, there were significant differences regarding basic characteristics and profiles of hormones, metabolism, and inflammation were observed between overweight and non-overweight patients with PCOS. Notably, the significantly differentially expressed inflammatory factors in overweight PCOS are strongly associated with their indicators of metabolic disorders, which may be related to the expression of IR-related proteins regulated by inflammatory factors.

### 3.1 Comparison of the basic characteristics and hormonal and metabolic profiles between overweight and non-overweight PCOS

We compared the baseline characteristics of overweight and non-overweight groups of women with PCOS. According to statistical results (continuous variables), compared with those in the non-overweight PCOS group, more advanced age and increased WHR were observed in overweight patients with PCOS. In the overweight PCOS group, the serum levels of LH, LH/FSH ratio, and ISI index were decreased, whereas the FBG levels, INS (namely INS_0min, INS_30min, INS_60min, INS_90min, INS_120min, and INS_180min), and the HOMA-IR and, IAUC indices were evidently increased. However, the clinical symptoms including paramenia, hirsutism, and acne and the serum levels of E_2_, T, DHEAS, and PRL were not significantly different between these two groups. Moreover, the overweight patients with PCOS had a higher rate of central obesity and acanthosis nigricans incidence and an incremental susceptibility to the family history of hypertension and diabetes compared with those in the non-overweight patients with PCOS. The detailed results are listed in **Tables 1**, **2**.

**Table 1:** Comparison of the baseline characteristics, clinical symptoms, endocrine parameters between overweight and normal weight PCOS patients.

Indexes	Overweight PCOS (BMI ≥ 24 kg/m^2^)	Non- overweight PCOS (BMI ≤ 23.9 kg/m^2^)	*P* value
**Personal information (246 vs 701)**			
**Age** (year)	26.56±4.97	25.02±4.92	0.0002
**Disease course** (year)	5.76±4.97	5.52±4.76	0.4993
**WHR**	0.86±0.05	0.76±0.03	0.0300
**Clinical symptoms**			
**Paramenia** (day) (246 vs 701)			
Length of menstrual cycle	76.88±66.75	71.76± 62.72	0.3306
Length of menstruation **Hirsutism** (F-G score) (66 vs 202)	6.27±3.68 9.12±5.35	6.01±2.57 8.67±4.78	0.3671* 0.4640
**Acne** (Rosenfield score) (80 vs 240)	1.29± 1.02	1.42±1.19	0.3850
**Sex hormone levels (246 vs 701)**			
**LH** (IU/L)	7.85±4.27	10.31±6.75	<0.0001*
**FSH** (IU/L)	6.75±4.23	7.01±4.17	0.5090
**LH/FSH**	1.23±0.72	1.55±1.43	<0.0001*
**E_2_** (pmol/L)	45.98±22.50	43.12±16.84	0.2090
**T** (μg/L)	0.65±0.35	0.71±0.41	0.1503*
**DHEAS** (μg/dL)	262.7±111.8	261.0±105.5	0.8982
**PRL** (μg/L)	20.00±41.38	20.01±62.28	0.9969*
**Serum biochemical levels (246 vs 701) **			
**FBG** (mmol/L) **Insulin_0min** (μIU/mL)	5.38±2.92 15.45±16.58	4.91±0.46 8.27±7.68	0.00758* 0.0001*
**Insulin_30min** (μIU/mL)	113.3±8.4806	78.7435±2.6437	<0.0001
**Insulin_60min** (μIU/mL)	131.0±79.54	75.07±56.27	<0.0001*
**Insulin_90min** (μIU/mL)	105.4±89.74	63.83±51.42	0.0001*
**Insulin_120min** (μIU/mL)	96.06±85.41	54.37±44.22	<0.0001*
**Insulin_180min** (μIU/mL)	40.25±46.44	22.75±29.22	0.0001*
**HOMA-IR**	3.17±18.55	0.92±1.64	<0.0016*
**IAUC**	247.2±214.8	126.1±183.5	<0.0001*
**ISI**	0.02±0.03	0.04±0.03	<0.0001

PCOS, polycystic ovary syndromes; SD, standard deviation; BMI, body mass index; WHR, waist-to-hip ratio; LH, luteinizing hormone; FSH, follicle-stimulating hormone; E_2_, estrogen; T, testosterone; PRL, prolactin; DHEAS, Dehydroepiandrosterone sulfate; FBG, Fasting Blood glucose; HOMA-IR, Homeostasis model assessment for insulin resistance; IAUC, Insulin area under the curve; ISI, insulin sensitivity index; T3, triiodothyronine; T4, thyroxine; TSH, thyrotropin. * Continuous variables were presented as mean ± SD. Satterthwaite was applied when the p value for F test (Equality of Variance test) <0.05.

**Table 2:** Comparison of the baseline characteristics, clinical symptoms, family history between overweight PCOS and non-overweight PCOS patients.

Indexes	Overweight PCOS (BMI ≥ 24 kg/m^2^)	Non- overweight PCOS (BMI ≤ 23.9 kg/m2)	*P* value
**Personal information (246 vs 701)**			
**Age**			0.3391
≤ 18 years	16 (6.50%)	48 (8.65%)	
> 18 years	230 (93.50%)	653 (93.15%)	
**Disease course**			0.9710
≤ 1 years	59 (23.98%)	173 (24.68%)	
>1, ≤ 10 years	154 (62.60%)	433 (61.77%)	
>10 years	33 (13.42%)	95 (13.55%)	
**WHR**			<0.0001
≤ 0.8	22 (9.14%)	365 (52.07%)	
> 0.8	224 (90.86%)	336 (47.93%)	
**Clinical symptoms** **Paramenia** (246 vs 701)	244 (99.19%)	688 (98.15%)	0.1413*
Length of menstruation			0.1162
21-28 days	22 (8.94%)	80 (11.41%)	
< 21, or > 28, ≤ 60 days	90 (36.59%)	280 (39.94%)	
> 60, ≤ 90 days	73 (29.67%)	215 (30.67%)	
> 90 days	61 (24.80%)	126 (17.97%)	
Oligomenorrhea	77 (31.30%)	231 (32.95%)	0.6341
**hirsutism** (66 vs 202)			0.8756
<7 (F-G score)	31 (46.97%)	100 (49.50%)	
>9 (F-G score)	35 (53.03%)	102 (50.50%)	
**Acne**(80 vs 240)			0.3740
0 (Rosenfield score)	20 (25.00%)	63 (26.25%)	
1 (Rosenfield score)	27 (33.75%)	74 (30.83%)	
2 (Rosenfield score)	26 (32.50%)	59 (24.58%)	
3 (Rosenfield score)	4 (5.00%)	29 (12.08%)	
4 (Rosenfield score)	3 (3.75%)	14 (5.83%)	
5 (Rosenfield score)	0 (0%)	1(0.42%	
**Acanthosis nigricans**			<0.0001
(76 vs 244)	46(60.53%)	82(33.61%)	
**Family history of diabetes, infertility, high blood pressure and menstrual irregularity**			
**Diabetes**			
(117 vs 271)	29(24.79%)	36(13.28%)	0.0054
**Infertility**			
(116 vs 272)	3 (2.59%)	4 (1.47%)	0.2273*
**High blood pressure**			
(115 vs 271)	48(41.74%)	79(29.15%)	0.0161
**Menstrual irregularities**)			
(119 vs 272)	15(12.61%)	30(11.03%)	0.6533

PCOS, polycystic ovary syndromes; BMI, body mass index; WHR, waist-to-hip ratio.

* Categorical variables were presented as the number of cases and column frequencies.

Fisher’s exact test was applied when number < 5/group.

### 3.2 Comparison of the serum inflammatory cytokine levels between overweight and non-overweight PCOS

**Figure 1:**
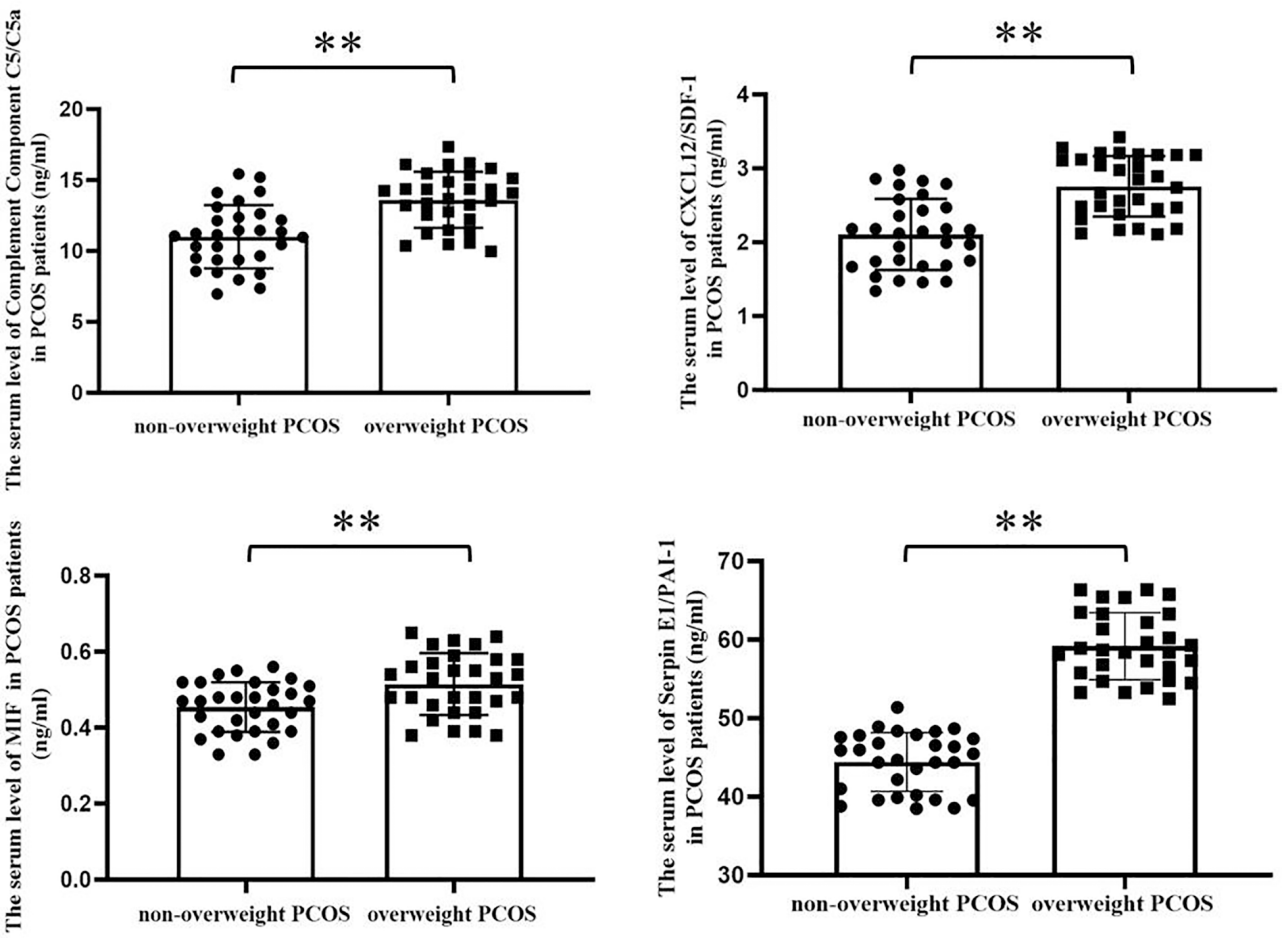
Serum levels of the significantly different inflammatory cytokines screened by protein microarray between overweight and non-overweight PCOS groups were verified by ELISA assay. As shown in the bar charts, the serum levels of Complement Component C5/C5a, CXCL12/SDF-1, MIF and Serpin E1/PAI-1 were obviously increased in overweight PCOS patients compared with the non-overweight PCOS patients (Complement Component C5/C5a, 11.010 ± 2.232 vs. 13.610 ± 1.977 ng/mL, p < 0.0001; CXCL12/SDF-1, 2.106 ± 0.482 vs. 2.758 ± 0.411 ng/mL, p< 0.0001; MIF, 0.454 ± 0.066 vs. 0.515 ± 0.081ng/mL, p = 0.0023; Serpin E1/PAI-1, 44.430 ± 3.751 vs 59.171 ± 4.274 p < 0.001, **, P < 0.01.).

Serum inflammatory cytokine protein microarray detection results showed that, the expression of inflammatory cytokines complement component C5/C5α, C‐X‐C motif chemokine ligand 12/stromal cell‐derived factor‐1 (CXCL12/SDF-1), macrophage migration inhibitory factor (MIF), and plasminogen activator inhibitor-1 (Serpin E1/PAI-1) were remarkably increased in overweight patients with PCOS compared with those in non-overweight group, **Table 3**. These findings were further verified using ELISA, **Figure 1**.

**Table 3:** Inflammatory cytokines with significantly different INT values between overweight PCOS and non- overweight PCOS detected by Proteome Profiler Human Cytokine Array Kit.

Index	Density INT/mm^2^		*P* value
	Overweight PCOS (BMI ≥ 24 kg/m^2^)	Non- overweight PCOS (BMI ≤ 23.9 kg/m^2^)	
**Reference Spots**	20228.5 ± 1100.5	20541.9 ± 1693.7	0.5434
**CCL5/RANTES**	22142.6 ± 1352.1	22106.6 ± 1243.1	0.9381
**CD40** **Ligand/TNFSF5**	17346.1 ± 3303.5	17567.0 ± 3041.4	0.8453
**complement component C5/C5α**	17622.4 ± 3074.9	11883.4 ± 7954.6	0.0143 *
**CXCL1/GROα**	8176.7 ± 4230.2	6642.9 ± 7317.5	0.4749
**CXCL12**	14851.7 ± 931.8	11651.4 ± 5820.2	0.0455 *
**ICAM-1/CD54**	21470.0 ± 1147.6	21668.3 ± 1732.7	0.7054
**IL-1ra/IL-1F3**	11180.2 ± 3212.4	10721.3 ± 1549.4	0.6120
**IL-8**	6129.5 ± 6536.4	18728.1 ± 32103.2	0.1433
**IL-13**	2040.7 ± 2751.8	1485.7 ± 2842.3	0.5789
**IL-16**	2040.7 ± 2751.8	3117.8 ± 3286.1	0.3228
**IL-18/IL-1F4**	7184.2 ± 1922.0	6717.7 ± 5589.1	0.7558
**MIF**	19369.3 ± 1100.1	17338.5 ± 3565.6	0.0432 *
**Serpin E1**	20749.5 ± 872.6	15630.2 ± 9424.2	0.0486 *

PCOS, polycystic ovary syndromes; SD, standard deviation; INT, intensity; BMI, body mass index; CCL5/RANTES, C-C motif chemokine 5; CD40 Ligand/TNFSF5, human leukocyte differentiation antigen CD40 ligand; CXCL1/GROα, melanoma growth stimulating factor/growth regulating oncogene α; CXCL12, C‐X‐C Motif Chemokine Ligand 12/stromal cell‐derived factor‐1; IL-1rα/IL-1F3, human interleukin 1 receptor α; IL-8, human interleukin 8; IL-13, human interleukin 13; IL-6, human interleukin 16; IL-18/IL-1F4, human interleukin 18; MIF, macrophage migration inhibitory factor; Serpin E1, Plasminogen Activator Inhibitor-1.

Continuous variables were presented as mean ± SD. Satterthwaite was applied when the p value for F test (Equality of Variance test) <0.05. * P-value < 0.05.

### 3.3 Correlations between highly expressed inflammatory cytokines and anthropometric measurements of overweight patients with PCOS

We analyzed the correlation between the special inflammatory cytokines (complement component C5/C5α, CXCL12/SDF-1, MIF, and Serpin E1/PAI-1) and anthropometric measurements (i.e., age, BMI, and WHR), sex hormones indices (i.e., LH and, LH/FSH), and metabolic indexes (i.e., FBG, INS, HOMA-IR, IAUC, and ISI) using Pearson correlation model. As shown in **Table 4**, complement component C5/C5α was positively correlated withINS_60min; CXCL12/SDF-1 was positively correlated with FBG, INS_0min, INS_60min, INS_90min, INS_120min and IAUC; MIF was positively correlated withINS_90min; and Serpin E1/PAI-1 was positively correlated withINS_0min, INS_30min, INS_60min, INS_90min, INS_180min, IAUC, and ISI. Collectively, the inflammatory factors were associated with glucose metabolism and IR in varying degrees.

**Table 4:** Correlation of inflammation cytokines and anthropometric measurements and biomarkers.

	complement component C5/C5α	CXCL12/ SDF-1	MIF	Serpin E1/ PAI-1
**Age**	-0.164	-0.015	0.369	-0.107
**BMI**	-0.089	-0.074	0.020	0.464
**WHR**	0.057	-0.043	-0.020	0.382
**LH**	-0.280	-0.238	0.008	-0.147
**LH/FSH**	-0.258	-0.078	0.175	0.071
**FBG**	-0.083	-0.207 *	0.254	0.400
**Insulin_0min**	0.162	0.094 *	0.142	0.562*
**Insulin_30min**	-0.072	0.195	0.192	0.579*
**Insulin_60min**	0.527 *	0.569*	0.462	0.615*
**Insulin_90min**	0.344	0.559*	0.501 *	0.518*
**Insulin_120min**	0.454	0.622*	0.313	0.383
**Insulin_180min**	0.306	0.220	0.379	0.517*
**HOMA_IR**	0.117	0.053	0.173	0.562
**IAUC**	0.375	0.573*	0.405	0.671**
**ISI**	-0.240	-0.380	-0.370	-0.685**

CXCL12/SDF-1, C‐X‐C Motif Chemokine Ligand 12/stromal cell‐ derived factor‐1; MIF, macrophage migration inhibitory factor; Serpin E1/PAI-1, Plasminogen activator inhibitor-1; BMI, body mass index; WHR, waist-to-hip ratio; LH, luteinizing hormone; FSH, follicle-stimulating hormone; FBG, Fasting Blood glucose; HOMA-IR, Homeostasis model assessment for insulin resistance; IAUC, Insulin area under the curve; ISI, insulin sensitivity index.

Pearson correlation analysis was performed between significantly elevated inflammatory factors (complement component C5/C5α, CXCL12/SDF-1, MIF and Serpin E1/PAI-1) with obvious anthropometric measurements and biomarkers. * P <0.05; ** P <0.01.

### 3.4 Correlations between inflammatory cytokines and IR-related regulatory proteins

**Figure 2:**
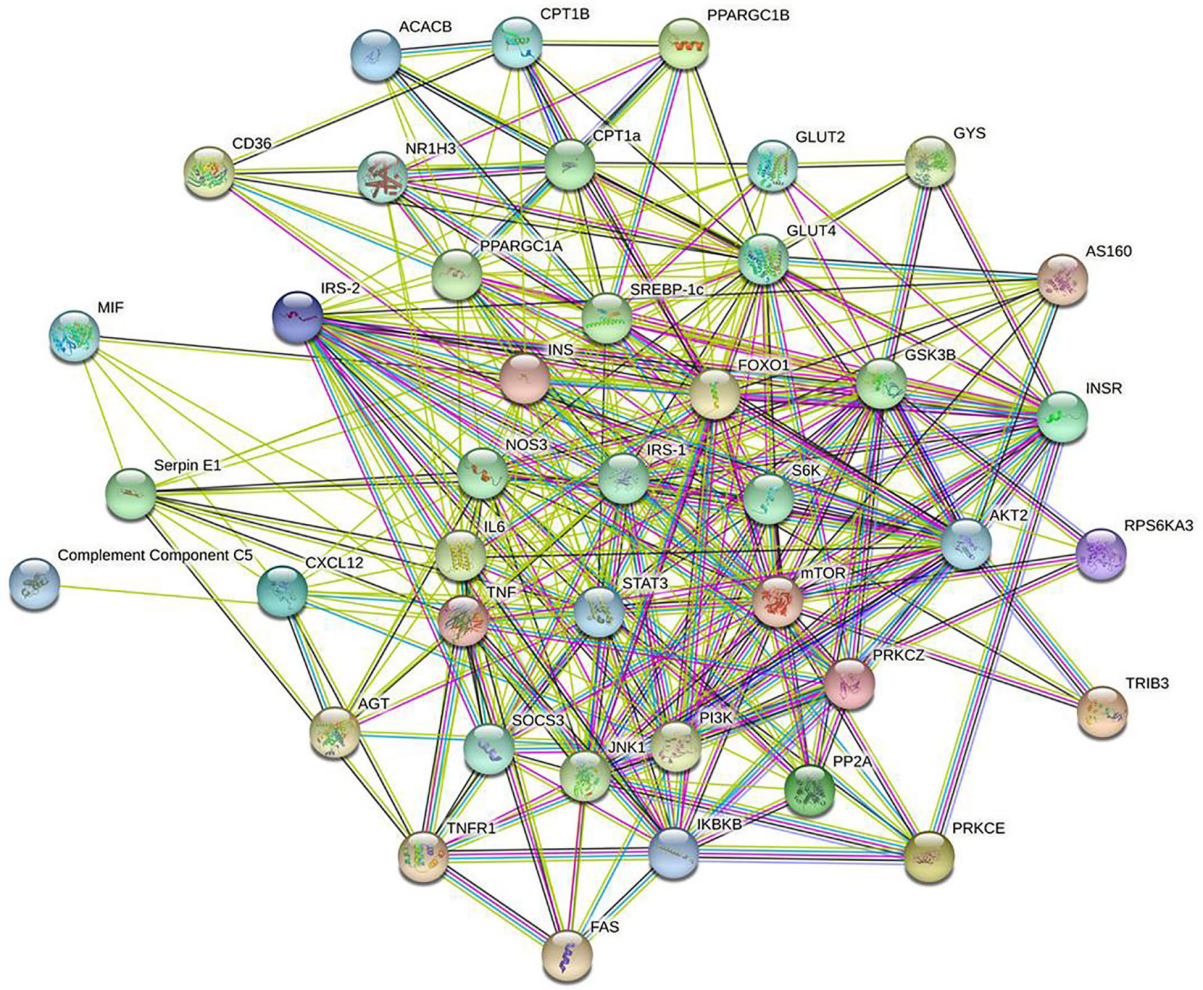
Interaction network of 4specific inflammatory factorsand 39 IR-related regulatory proteinsbuild by String online server.

**Figure 3:**
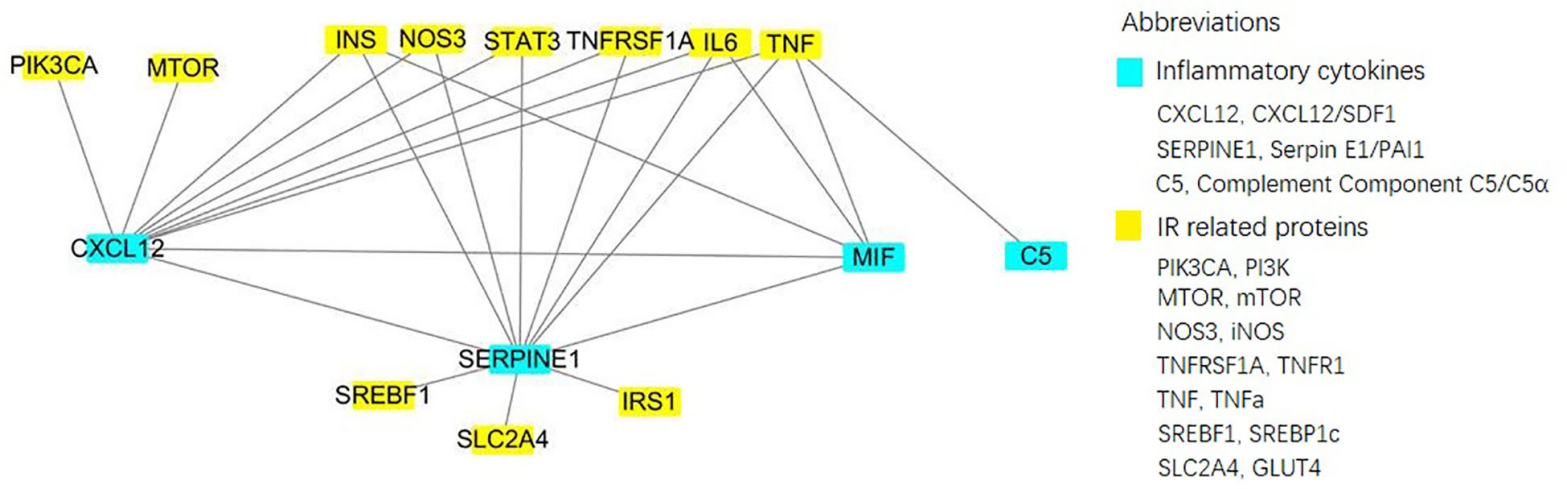
Direct interactionof 4 specific inflammatory factorsand 11 key IR-related regulatory proteinsbuild by Cytoscape online server.

**Figure 4:**
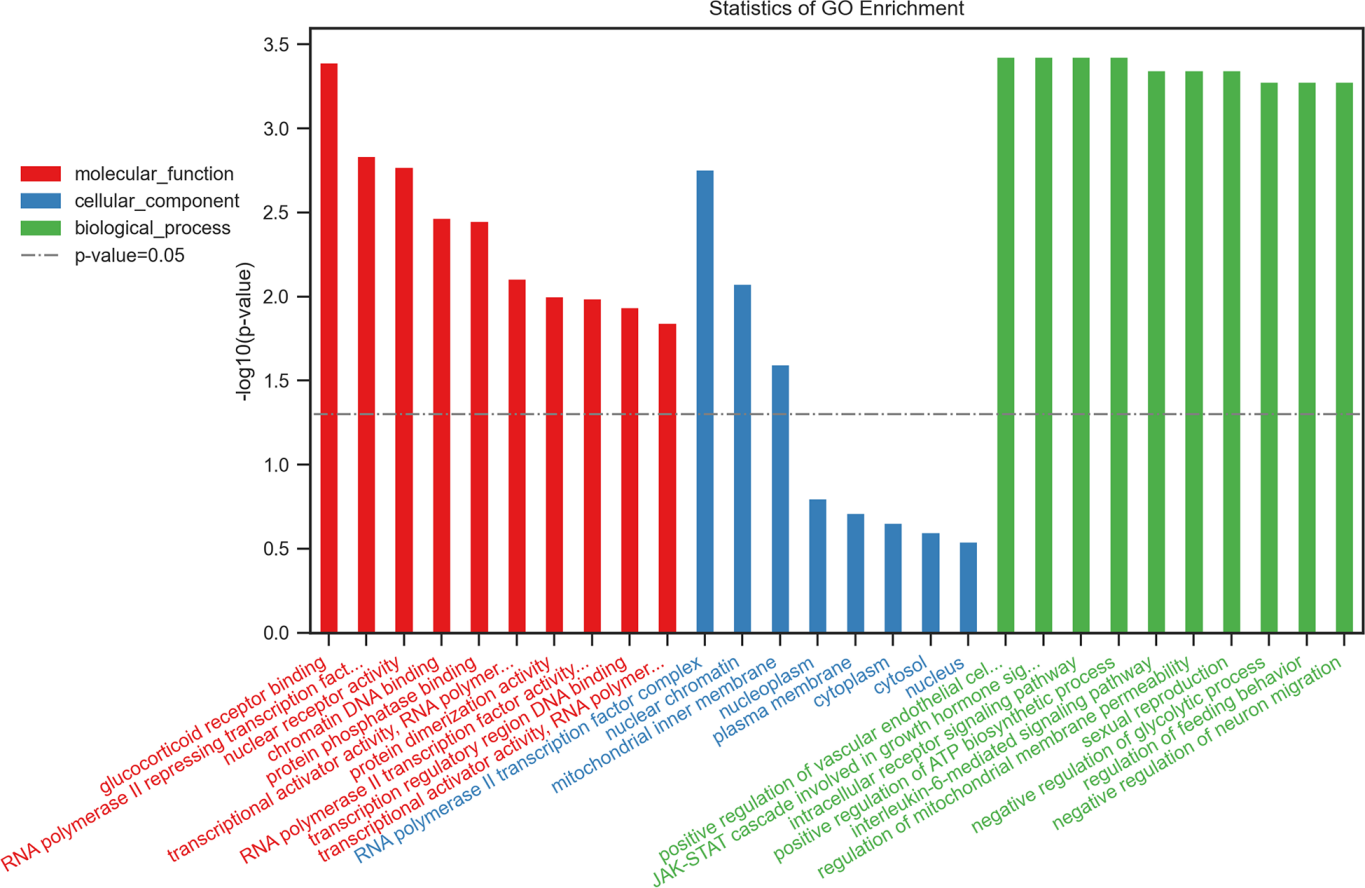
Top 10 of the GO-enrichment analysis for the biological process, molecular functions, cellular components of 15 proteins including 4 inflammatory cytokinesand 11 key IR-related regulatory proteinsbuild byOECloudonline server.

**Figure 5:**
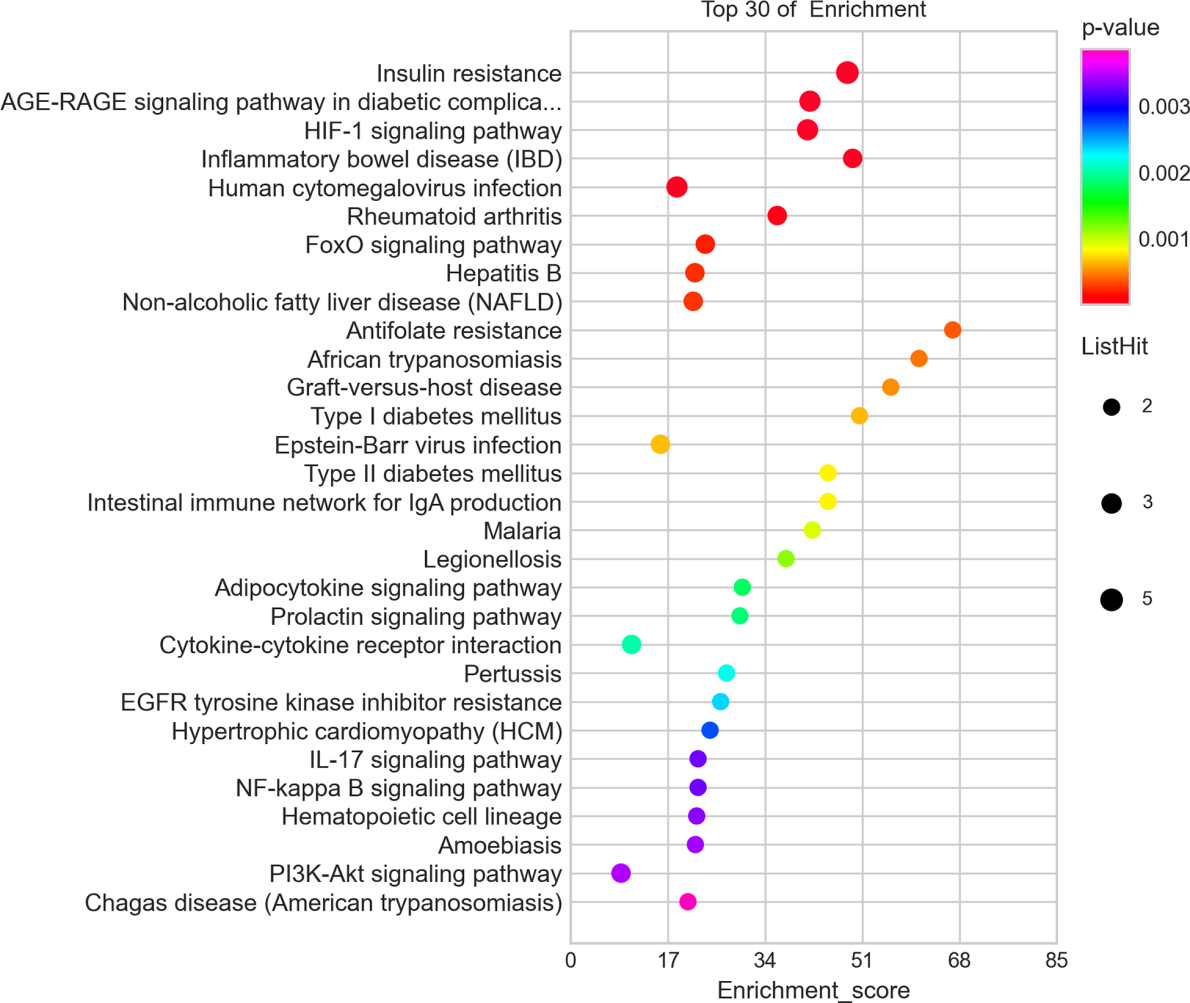
Top 30 of the KEGG-enrichment analysisof 15 proteins including 4 inflammatory cytokinesand 11 key IR-related regulatory proteinsbuild byOECloudonline server.

IR-related proteins were acquired from the KEGG database, and 39 related regulatory proteins in the IR signaling pathway were screened in our research, including INS (K04526), INSR (K04527), IL-6 (K05405), TNFα (K03156), AGT (K09821), TNFR1 (K03158), CD36 (K06259), STAT3 (K04692), SOCS3 (K04696), JNK1 (K04440), IKKβ (K07209), NFκB (K02580), ACCβ (K01946), IRS-1 (K16172), IRS-2 (K07187), iNOS (k13242), CPT1β (K19523), PP2A (K17605), PI3K (K00922), PKC (K18952), TRIB3 (K19518), ISPK-1 (K04373), S6K (K04688), AKT2 (K04456), mTOR (K07203), GSK-3 (K03083), GS (K00693), AS160 (K17902), GLUT4 (K07191), GLUT2 (K07593), SREBP-1c (K07197), FOXO1 (K07201), PGC-1α (K07202), PGC-1β (K17962), SREBP-1c (k07197), FAS (K11533), PKC-ϵ (KPRKCE), CPT1α (K08765) and LXR (K08536). Network databases for predicting PPIs with confidence score ranges were used to explore the correlation between the 4 specific inflammatory cytokines and 39 IR-related regulatory proteins. As indicated by the STRING analysis, each inflammatory cytokine was directly or indirectly connected with IR-related regulatory proteins (**Figure 2**). Furthermore, the direct correlations between these two kinds of proteins were analyzed using Cytoscape, and 11 key IR-related regulatory proteins, namely PI3K, mTOR, INS, iNOS, STAT3, TNFR1, interleukin (IL)-6, tumor necrosis factor (TNF) α, SREBP-1c, GLUT4, and IRS-1, were screened (**Figure 3**). Furthermore, GO-enrichment analysis for the biological processes, molecular functions, and cellular components and KEGG-enrichment analysis of these 15 proteins (i.e., 4 inflammatory cytokines and 11 key IR-related regulatory proteins) were performed using OECloud online server. Consequently, we found multiple biological processes between inflammation and IR (P < 0.01). The top five of these processes were the JAK-STAT cascade involved in the growth hormone signaling pathway (GO:0060397), intracellular receptor signaling pathway (GO:0030522), positive regulation of vascular endothelial cell proliferation (GO:1905564), positive regulation of ATP biosynthetic process (GO:2001171) and regulation of mitochondrial membrane permeability (GO:0046902). The top five molecular functions terms (P < 0.01) included glucocorticoid receptor binding (GO:0035259), RNA polymerase II repressing transcription factor binding (GO:0001103), regulation of mitochondrial nuclear receptor activity (GO:0001103), chromatin DNA binding (GO:0031490), and protein phosphatase binding (GO:0019903). The main cellular component terms (P < 0.01) were RNA polymerase II transcription factor complex (GO:0090575), nuclear chromatin (GO:0000790), mitochondrial inner membrane (GO:0005743), nucleoplasm (GO:0005654), and plasma membrane (GO:0005886); **Figure 4**. In addition, these proteins were further mapped to several KEGG pathways with P < 0.01, including IR (has04931), AGE-RAGE signaling pathway in diabetic complications (hsa04933), HIF-1 signaling pathway (has04066), FoxO signaling pathway(hsa04068), IL-17 signaling pathway(hsa04657), NF-kappa B signaling pathway(has04064), and PI3K-Akt signaling pathway (has04151). Detailed results are presented in **Figure 5** and **Supplementary Table 2–4**.

## 4 Discussion

PCOS is a common endocrine-metabolic disorder in women of childbearing age; its prevalence has been increasing worldwide (17). An incremental prevalence of PCOS has been reported in overweight women; this prevalence is estimated to be 14-75% among different ethnic groups (18), and the probability of PCOS reportedly increases by 9.2% per one unit increase in BMI (19). In the present study, A 947 women with PCOS, 25.98% were overweight (defined as BMI ≥ 24 kg/m^2^), among which 90.86% of cases presented as central obesity (defined as WHR > 0.8). On conducting statistical analyses, no apparent difference was observed between the overweight and non-overweight PCOS groups in terms of paramenia, acne, and hirsutism. However, acanthosis nigricans was reported to occur frequently in women with hyperandrogenism and diabetes mellitus (20). Meanwhile, essential diagnostic investigations in PCOS remain controversial. An LH/FSH ratio >2 or 2.5 has been considered as “gold standard” in PCOS diagnosis for a long time. Recognizing the role of LH and ratio of LH/FSH and evaluating the usefulness of gonadotropin in PCOS diagnosis is an intriguing challenge (21). A clinical study in Italy showed that elevated LH concentration and LH/FSH ratio are endocrine characteristics of PCOS and are closely related to BMI (22); nevertheless, in another clinical study in Saudi Arabia, researchers determined that BMI was not correlated with increased LH/FSH ratio (23). Notably, our study found that low gonadal levels, including decreased LH concentrations and LH/FSH ratio, in overweight PCOS were more pronounced compared with those in non-overweight PCOS; this result, is consistent with those of previous studies (24, 25). Obesity can cause decreased LH pulse amplitude; this has been demonstrated in animal studies in which female rhesus monkeys on a high-fat diet showed reduced LH pulse amplitude compared to that of those on a normal diet (26). Additionally, the accelerated metabolism and clearance of LH caused by increased sialic acidification in patients are key reasons for the decreased level of LH in obese patients with PCOS (27, 28). In general, as the obesity levels of PCOS included in each group of studies are not consistent, different LH levels and LH/FSH ratios will naturally be obtained; thus, further clinical research is warranted. Moreover, genetic predisposition is partially attributed to the etiology of PCOS. Our results showed the proportion of family hypertension and diabetes in overweight PCOS was increased. Therefore, we speculated that familial hypertension and diabetes are important genetic susceptibility factors affecting PCOS development.

Patients with PCOS, especially those comorbid with overweight, are closely associated with extreme IR. Approximately 50-70% of obese women with PCOS are insulin resistant, although the accuracy of this figure was undermined by frequent referral bias (29). Consistently, the serum biochemical indices including FBG, INS, HOMA-IR, and IAUC in overweight PCOS were significantly increased and ISI was decreased compared with those in the non-overweight PCOS in our study. These results suggested that there was obvious IR in overweight patients with PCOS. Insulin has physiological effects such as mediating the uptake and utilization of glucose by tissue cells, promoting lipid and protein synthesis, and inhibiting protein decomposition. Fat accumulation in metabolic dysfunction patients could reduce the uptake and utilization of glucose by peripheral tissues by increasing liver gluconeogenesis, decreasing hepatic insulin clearance, and ultimately leading to IR (30). IR stimulates food intake, increases fat accumulation, and thus exacerbates/causes overweight or even obesity, creating a vicious circle between overweight and IR among patients with PCOS (31). However, the underlying pathogenesis of IR in PCOS has not yet been fully elucidated. Recently, the chronic inflammation theory has gained attention in PCOS pathogenesis research since Kelly et al. first reported significantly elevated C-reactive protein (CRP) concentrations in patients with PCOS and indicated low-grade chronic inflammation to be a novel mechanism of coronary heart disease and type 2 diabetes in those women (32).

In patients with PCOS, low-grade chronic inflammation is a long-standing immune inflammation with a lesser degree of inflammatory microenvironment than that of acute inflammation caused by a bacterial or viral infection. Currently, chronic low-grade inflammation states and inflammatory markers have emerged as noteworthy contributors to the pathogenesis of PCOS, and the inherent interaction between chronic low-grade inflammation and IR contributes to the development of PCOS. As per known data, levels of inflammatory markers such as IL-18, TNF-α, IL-6, white blood cell count, monocyte chemoattractant protein-1, and macrophage inflammatory protein-1α are higher in patients with PCOS than in the age- and BMI-matched healthy women (31). Notably, the levels of four inflammatory cytokines, including complement component C5/C5α, CXCL12/SDF-1, MIF, and Serpin E1/PAI-1, were significantly elevated in overweight PCOS compared with those in non-overweight PCOS based on our Protein microarray analysis, and this is the first report on the correlation different inflammatory cytokines’ correlation with the BMI of PCOS. Only a few studies have reported the association of increased complement component C5/C5α and CXCL12/SDF-1 levels among overweight PCOS. Lewis et al. observed the stimulation of the complement system through to the terminal pathway including complement component C5/C5α cleavage and initiate the construction of the membrane-attacking complex and its fluid-phase by-product, the terminal complement complex among patients with PCOS having IR (33). CXCL12/SDF-1 was first identified by Bleul et al. in 1996 as a compelling leukocyte attractant in the supernatants of murine bone marrow cell lines and demonstrated to appeal to both human lymphocytes and monocytes (34). CXCL12/SDF-1 has been reported both as a stimulator and an antagonist of several perspectives of inflammatory response in type 2 diabetes and its complications and CXCL12/SDF-1might be a promising treatment target to improve islet engraftment (35). To the best of our knowledge, this study is the first to report the potential pathogenic role of CXCL12/SDF-1 in PCOS and incremental serum levels of CXCL12/SDF-1 in overweight patients with PCOS. MIF is a proinflammatory cytokine encoded within a functionally polymorphic genetic locus, the pro-inflammatory nature of which is often associated with the spread of inflammation and autoimmune diseases. Moreover, MIF has other special functions unrelated to the immune system, such as supporting post-translational modifications of insulin (36, 37). Serpin E1/PAI-1is a key regulator of fibrinolysis and has higher circulating levels in PCOS (38), which has also been positively related to circulating insulin levels, especially in the presence of obesity. Several studies have investigated Serpin E1/PAI-1 activity and its association with PCOS; however, the results remain controversial (39, 40). Based on our findings, the aforementioned four inflammatory factors were associated with glucose metabolism and IR in varying degrees. Additionally, as per our bioinformatics analysis, Serpin E1/PAI-1 and CXCL12/SDF-1were directly correlated with several IR-related proteins, such as INS, NOS3, STAT3, TNFR1, IL-6, and TNFα. MIF was directly correlated with INS, IL-6, and TNFα, and complement component C5/C5α was directly correlated with TNFα. Furthermore, Serpin E1/PAI-1, CXCL12/SDF-1, and MIF were directly correlated. IL-6 and TNFα are important inflammatory cytokines, and key proteins in IR-related signaling pathways. Therefore, we speculate that inflammatory factors can induce IR and further promote the production of other inflammatory factors, thereby forming a cascade reaction that leads to IR. These processes involve several biological functions and signal transduction, including the JAK-STAT cascade, ATP biosynthetiesis, and HIF-1 signaling.

We believe that our results offer new insights into the potential physical pathogenesis involved in PCOS and might unravel the significance of close monitoring of the proposed inflammatory cytokines (i.e., CXCL12/SDF-1, Serpin E1/PAI-1, and MIF) for women with overweight. Our findings underscore the importance of elucidating the underlying role of these inflammatory cytokines in the physiopathology and progression of PCOS, especially among women comorbid with overweight. Our study has some limitations. First, this is a hospital-based retrospective cross-sectional study conducted in a Medical University affiliated comprehensive hospital with a population that has a low risk for PCOS. Second, as this study included an ethnically homogeneous Chinese population with a predominant Han ethnicity, the extent to which our findings can be generalized to other populations remains undetermined. Lastly, the statements on the causality of the associations and implications for the following clinical management were limited given the observational nature of the study.

## 5 Conclusion

The findings of our study indicated that overweight patients with PCOS exhibit evident characteristics of low gonadal levels (i.e., decreased LH levels and LH/FSH ratio), IR, and high inflammation levels. The inflammatory cytokines CXCL12/SDF-1, Serpin E1/PAI-1, and MIF increased significantly among overweight PCOS patients, and these cytokines might lead to IR through various biological functions and signal transduction. These findings may provide some guidance for the clinical diagnosis and treatment of PCOS.

## Data availability statement

The datasets presented in this study can be found in online repositories. The names of the repository/repositories and accession number(s) can be found in the article/**Supplementary Material**.

## Ethics statement

The studies involving human participants were reviewed and approved by Ethics Committee of Shanghai Changhai Hospital. Written informed consent to participate in this study was provided by the participants’ legal guardian/next of kin.

## Author contributions

JY, YZ, and JD designed the study, collected the data, conducted statistical analyses and drafted the original version of the manuscript. DZ offered intellectual support for the framework of the study. CY and HH are guarantors of this manuscript and supervised the data collection. All authors contributed to the article and approved the submitted version.
