# Supplementary material for: Characteristics and possible mechanisms of metabolic disorder in overweight women with polycystic ovary syndrome
Source: Front Endocrinol (Lausanne). 2023 Jan 12;13:970733. doi: 10.3389/fendo.2022.970733 (PMC9878688; doi:10.3389/fendo.2022.970733)
Supplement: Supplementary file 3 [file Table_1.docx]

**Supplementary Table 1. Kits used in this research**

| Kits name | Source | | Catalogue No. |  |
| --- | --- | --- | --- | --- |
| Chemiluminescence particle immunoassay kits | | | | |
| LH Reagent Kit | Abbott Trading (Shanghai) Co., LTD | | Lot. 39367UD02 | |
| FSH Reagent Kit | Abbott Trading (Shanghai) Co., LTD | | Lot. 30921UD02 | |
| T Reagent Kit | Abbott Trading (Shanghai) Co., LTD | | 20162404132 | |
| E2 Reagent Kit | Abbott Trading (Shanghai) Co., LTD | | Lot. 42387UD01 | |
| PRL Reagent Kit | Abbott Trading (Shanghai) Co., LTD | | Lot. 41656UD01 | |
| DHEAS Reagent Kit | Abbott Trading (Shanghai) Co., LTD | | Lot. 41024UD01 | |
| Hexokinase kit | |  |  | |
| FBG Reagent Kit  Electrochemiluminescence kits | Box Shenggong (Beijing)Technology Co., LTD | | Lot. AKSU061 | |
| INS Reagent Kit  Proteome Profiler Human Cytokine Array Kit | Roche Diagnostics (Shanghai) Co., LTD  R&D Systems | | lot. 20162404356  Lot. ARY005B | |
| ELISA kits  complement component C5/C5α  CXCL12/SDF-1  MIF  Serpin E1/PAI-1 | Beyotime systems  Beyotime systems  Beyotime systems  Beyotime systems | | Product No.PC094  Product No.PC205  Product No. PM715  Product No. PP785 | |
